# Mineralocorticoid Excess or Glucocorticoid Insufficiency: Renal and Metabolic Phenotypes in a Rat Hsd11b2 Knockout Model

**DOI:** 10.1161/HYPERTENSIONAHA.115.05262

**Published:** 2015-08-12

**Authors:** Linda J. Mullins, Christopher J. Kenyon, Matthew A. Bailey, Bryan R. Conway, Mary E. Diaz, John J. Mullins

**Affiliations:** From the Molecular Physiology Laboratory, University of Edinburgh/BHF Centre for Cardiovascular Science, Queen’s Medical Research Institute, Edinburgh, United Kingdom.

**Keywords:** knockout rat, mineralocorticoid excess syndrome, apparent, rat Hsd11b1 protein, rat Hsd11b2 protein, zinc-finger nuclease

## Abstract

Obesity and hypertension are 2 major health issues of the 21st century. The syndrome of apparent mineralocorticoid excess is caused by deficiency of 11β-hydroxysteroid dehydrogenase type 2 (Hsd11b2), which normally inactivates glucocorticoids, rendering the mineralocorticoid receptor aldosterone–specific. The metabolic consequences of Hsd11b2 knockout in the rat are investigated in parallel with electrolyte homeostasis. Hsd11b2 was knocked out, by pronuclear microinjection of targeted zinc-finger nuclease mRNAs, and 1 line was characterized for its response to renal and metabolic challenges. Plasma 11-dehydrocorticosterone was below detection thresholds, and Hsd11b2 protein was undetected by Western blot, indicating complete ablation. Homozygotes were 13% smaller than wild-type littermates, and were polydipsic and polyuric. Their kidneys, adrenals, and hearts were significantly enlarged, but mesenteric fat pads and liver were significantly smaller. On a 0.3% Na diet, mean arterial blood pressure was ≈65 mm Hg higher than controls but only 25 mm Hg higher on a 0.03% Na^+^ diet. Urinary Na/K ratio of homozygotes was similar to controls on 0.3% Na^+^ diet but urinary albumin and calcium were elevated. Corticosterone and aldosterone levels showed normal circadian variation on both a 0.3% and 0.03% Na^+^ diet, but plasma renin was suppressed in homozygotes on both diets. Plasma glucose responses to an oral glucose challenge were reduced despite low circulating insulin, indicating much greater sensitivity to insulin in homozygotes. The rat model reveals mechanisms linking electrolyte homeostasis and metabolic control through the restriction of Hsd11b1 substrate availability.

Despite advances in our understanding of genetic and environmental factors contributing to essential hypertension and the metabolic syndrome (which may be a confounding issue), the underlying pathophysiology is often unknown. Rare monogenic hypertensive disorders have revealed that proteins involved with regulation of mineralocorticoid secretion and activity, which ultimately affect sodium balance are key to the maintenance of blood pressure homeostasis.^1–3^ Many of these are remediable with glucocorticoid treatment. Conversely, associated diseases of metabolic syndrome (including visceral obesity, insulin resistance, and type 2 diabetes mellitus) involve systems modulated by glucocorticoid activity,^4^ but the mechanisms linking electrolyte homeostasis and metabolic control have not been investigated in parallel. We have developed a new rodent model of the syndrome of apparent mineralocorticoid excess (SAME; OMIM No. 218030) to study glucocorticoid–mineralocorticoid cross-talk in more detail.

SAME is characterized by a deficiency of the enzyme 11β-hydroxysteroid dehydrogenase type 2 (Hsd11b2), which inactivates glucocorticoid hormone (cortisol and corticosterone) by oxidation to 11-dehydro derivatives (cortisone and 11-dehydrocorticosterone).^5^ Normally, Hsd11b2 allows aldosterone, a hormone that circulates at much lower concentrations than glucocorticoids, to bind to the mineralocorticoid receptor. In the absence of Hsd11b2 activity, glucocorticoids bind inappropriately to mineralocorticoid receptor evoking excess mineralocorticoid activity (ie, sodium and water retention and potassium excretion) in tissues, such as kidney, colon, and sweat glands. Patients with SAME also present with a suppressed renin–angiotensin–aldosterone system, short stature, failure to thrive, polydipsia, polyuria, and other renal pathologies.^6^

Theoretically, Hsd11b2 should also limit access to glucocorticoid receptors (GR). However, although there are precedents for Hsd11b2-mediated protection of GR in lower vertebrates^7^ and placenta,^8^ GR expression is more widespread than Hsd11b2. More important are the potential secondary consequences of failure to synthesize 11-dehydro derivatives. Hsd11b1, (the type 1 isozyme), regenerates active glucocorticoid from cortisone and 11-dehydrocosticosterone at peripheral targets, such as liver and adipose tissues with significant metabolic consequences. For example, mice with transgenic overexpression of Hsd11b1 in adipose tissue are obese and hypertensive.^9^ It follows that in the absence of Hsd11b2, Hsd11b1 will also be inactive through lack of substrate, causing a syndrome of glucocorticoid insufficiency.

To study these glucocorticoid- and mineralocorticoid-related phenotypes, we have used zinc-finger nuclease (ZFN) technology^10,11^ to generate a knockout rat model of SAME. ZFNs are designed to generate a specific double-strand break in the gene of interest. This is repaired by nonhomologous end joining, which is prone to errors and frequently leads to the insertion or deletion of nucleotides. Targeted deletions were introduced into exon 2 of the *Hsd11b2* gene, leading to interruption of the gene product.

Hsd11b2 knockout rats demonstrate many of the symptoms of SAME, including reduced size, polydipsia, polyuria, and salt-sensitive hypertension, which were previously observed in an Hsd11b2 knockout mouse generated by standard gene–targeting techniques.^12–17^ Although rats did not develop the profound salt wasting and progressive loss of renal medulla seen in older knockout mice, significant renal damage was observed at 9 months of age. Hsd11b2 knockout rats also revealed protective effects relating to glucose handling and insulin sensitivity.

## Methods

Detailed methods section is available in the online-only Data Supplement.

### Statistical Analyses

Data are presented as the mean±SEM. Variables (genotype and diet) were compared by performing 2-way ANOVA using Prism6. Mean values were compared using the Student *t* test. A *P*<0.05 was considered to be statistically significant.

## Results

ZFNs were designed to bind specifically to exon 2, near to the sequence encoding the nicotinamide adenine dinucleotide–binding site^18^ (Figure S1A in the online-only Data Supplement). Targeting was achieved by pronuclear microinjection of ZFN mRNAs into single-cell embryos (Table S1). Injection of ZFN mRNAs at 10 ng/µL was toxic to the cells, as pup numbers were dramatically reduced. Targeting was achieved using ZFN mRNAs at a concentration of 3.125 ng/µL. Of 48 pups born, 4 targeted progeny were identified (Table S1) using polymerase chain reaction (PCR) primers designed to produce a 332-bp product spanning the target site (Table S2). PCR products were screened for mismatch and cleavage with surveyor nuclease. Subsequent sequencing of subcloned PCR products revealed the extent of insertions and deletions.

Two founders carried small deletions (1 and 16 bp), whereas 1 carried a single base-pair insertion at the target site. Founder no. 6 contained multiple-targeted alleles (Figure S1B), suggesting that the ZFNs remain active in the embryo beyond the 2-cell and 4-cell stages of cell division. Deletions of 1, 4, and 16 bp brought a TGA nucleotide triplet stop codon into frame (Figure S1B), resulting in truncation of the protein. The 123 bp deletion removed the 3′ end of exon 2 and the first 16 bp of intron B. If transcription and translation continued beyond this point, then a TAG nucleotide triplet stop codon would be brought into frame. The 332 bp deletion removed the entire second exon (extending 84 bp upstream and 37 bp downstream into the flanking introns), which would minimally remove the nicotinamide adenine dinucleotide–binding site from any translated protein product. All alleles were segregated in the progeny of founders by mating with wild-type (WT) F344IcoCrl females. Although preliminary phenotypic analysis indicated that at least 2 lines were polydipsic and polyuric, only the line carrying a 123 bp deletion was characterized in detail.

### Phenotypic Characterization of *Hsd11b2* Knockout Animals: Morphology

Homozygous animals were indistinguishable from littermates at weaning, but were significantly smaller than heterozygous or WT animals at 12 weeks of age. Body weights of male *Hsd11b2*^−/−^ (Hsd2KO) animals at 14 weeks old were 13% lower than age-matched WT animals (276.7±4.1 [n=7] versus 319.8±6.1g [n=9]; *P*<0.05). Female Hsd2KO animals (12 weeks old) were 12.5% smaller than age-matched controls (166.2±2.6 [n=5] versus 190.5±2.5 g [n=4]). Hsd2KO animals had significantly larger kidneys, adrenals, and hearts than age-matched control animals, whereas liver and mesenteric fat pads were significantly smaller (Figure S2).

Kidneys of young Hsd2KO animals (9 weeks of age) showed no gross pathological changes. Homozygotes exhibited weak immunoreaction with anti-Hsd11b2 antibody, (Figure S3C and S3E [WT] versus S3G and S3I [Hsd2KO]). Reverse transcription PCR primers were therefore designed, which spanned all introns between exons 2 and 5, to check for any aberrant transcripts from possible splice variants (Table S3). Although transcripts were observed, there were clear signs of aberrant/reduced splicing of the 123-bp–deleted gene transcript (Figure S4). For example, using primers spanning exons 2 to 3 (flanking the 123 bp deletion), a ≈40-bp reverse transcription PCR product was observed rather than the predicted 147 bp product (Figure S3A, lanes 1–4). A possible cryptic splice donor was identified, 2 bases upstream from the TAG stop codon that had been predicted to truncate the protein product. This would account for the reverse transcription PCR results and would lead to the production of a protein lacking 36 amino acids. Western analysis clearly demonstrated a complete lack of the ≈40-kD WT protein (Figure S5) suggesting that no stable full-length protein is made from the aberrantly spliced transcripts. Hsd11b2 converts corticosterone (B) to 11- dehydrocorticosterone (A) and plasma A was below detection threshold in homozygotes, indicating loss of Hsd11b2 activity (Figure S6).

Despite the increased kidney size, nephron counts were not different between genotypes (data not shown). Kidneys of 6-month-old null males showed subtle increases in fibrosis compared with controls (Figure S7). Kidney injury molecule 1 (Kim-1) antibody staining was significantly increased, and affected tubules were often associated with reactive macrophages as shown by the Cd68 macrophage marker, ED-1 staining (Figure S7). Dissection of older adults (9–12 months) revealed clear signs of kidney damage, with multifocal accumulation of amorphous acidophilic material expanding the Bowman space of some glomeruli (glomerular sclerosis) and tubules on hematoxylin–eosin (H&E) staining (Figure S8). There was clear interstitial expansion with lymphoplasmacytic inflammation and chronic fibrosis. The arcuate artery showed fibrinoid necrosis and there were signs of arteritis, tubule distension, and tubule degeneration (epithelial cells sloughed off). Crystalline calcium deposits were also observed (Figure S8).

Patients with SAME exhibit hypertrophy and hyperplasia of the distal convoluted tubules. Therefore, serial sections of kidneys from 6-month-old rats were immunostained with anti-Slc12a3/NCC and anti-Hsd11b2 antibodies to distinguish the distal collecting tubule DCT1 from the more distal DCT2, connecting tubule and collecting ducts. Slc12a3/NCC staining was broadly similar between WT and Hsd2KO animals (Figure S3B, S3D versus S3F, S3H). Areas of colocalization of immunostain in serial sections (eg, Figure S3D and S3E), confirmed an overlap of expression along the DCT. A trend toward hyperplasia of DCT1 was suggested in Slc12a3/NCC-expressing tubules (Figure S3A), which reached significance in Hsd11b2-expressing tubules. (No tubule hyperplasia was demonstrated in proximal tubules, using anti-Hsd11b1 antibody.) Unlike patients with SAME, only hyperplasia and not hypertrophy of the DCT or connecting tubule/collecting ducts was observed in the young adult rats.

**Figure 1.**
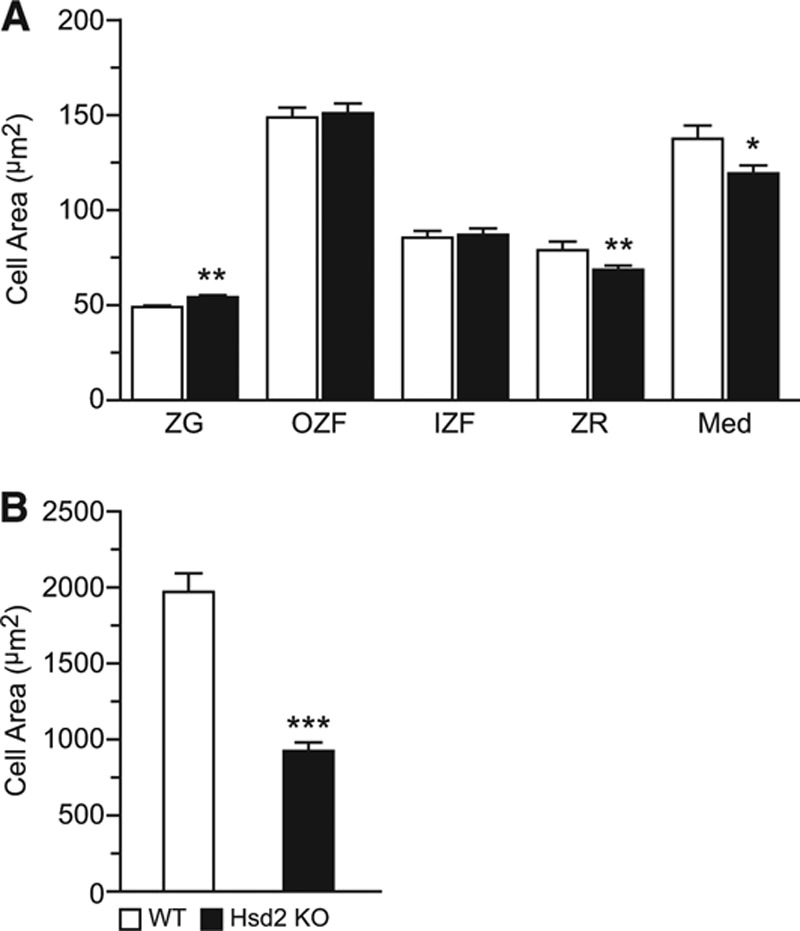
Morphometric analysis of (**A**) adrenal zonal cell areas and (**B**) mesenteric fat cell areas. Med indicates medulla; WT, wild-type; ZF, zona fasciculata; ZG, zona glomerulosa; and ZR, zona reticularis. *P<0.05; **P<0.01; ***P<0.001.

Enlargement of the adrenal gland in Hsd2KO was unexpected (Figure S2). Histological examination of H&E stained sections (Figure S9A and S9B) suggested this was because of increased width of the zona fasciculata though morphological measurements suggested that cell hypertrophy was not involved. However, zona glomerulosa cells were marginally bigger, whereas zona reticularis and medullary cells were smaller (Figure 1A). Expression of Hsd11b2 was confirmed by immunohistochemistry in the zona fasciculata and zona reticularis of WT but not Hsd2KO adrenals (Figure S9C–S9F).

Heart tissue sections of 6-month-old rats were stained with Masson Trichrome and with anti–ED-1 antibody. Multifocal mild subendocardial replacement fibrosis of the ventricular walls was observed. All homozygotes showed a mild but significant increase in macrophage numbers (32.8±3.0 per field [×20 magnification] versus 22±1.3 control; *P*<0.05) and mild multifocal increased fibrosis of the papillary muscles compared with controls (Figure S10). The dramatic reduction in mesenteric fat pads in the Hsd2KO animals was because of significantly reduced adipocyte size, suggesting decreased fat storage (Figure 1B and S9G–S9H). No sign of increased inflammatory cell infiltration was observed.

### Blood Pressure

Older Hsd2KO animals (9–12 months of age) exhibited adverse effects of chronic hypertension, including pulmonary congestion and enlarged hearts with concentric cardiac hypertrophy and congested right atria. Two Hsd2KO females had massively enlarged mesenteric arteries with marked expansion of the tunica media, suggestive of chronic active pan-arteritis (Figure S8G–S8I).

**Figure 2.**
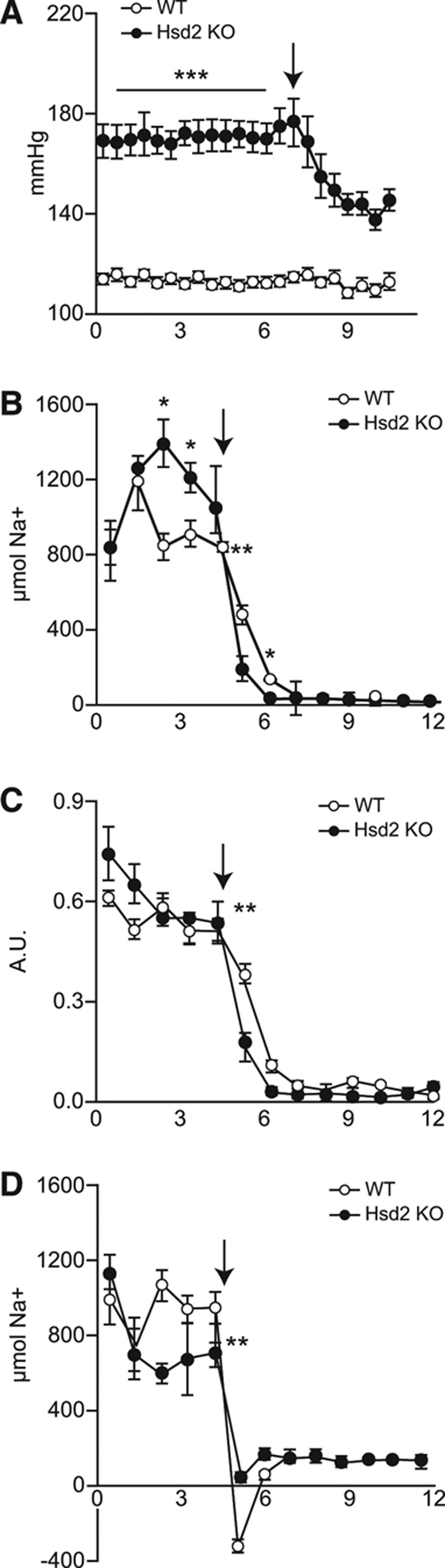
Graphs showing daily (**A**) MABP; (**B**) urinary Na; (**C**) urinary Na/K ratio; (**D**) average Na balance of wild-type (WT; open circle) and Hsd2KO (closed circle) during transition from 0.3% Na diet to 0.03% Na diet (arrowed; *P<0.05; **P<0.01; ***P<0.001).

Baseline telemetric measurements revealed that on a 0.3% Na diet Hsd2KO animals were severely hypertensive with a mean arterial blood pressure of 170.5 mm Hg (compared with WT–113.5 mm Hg). Change to a 0.03% Na diet reduced mean arterial blood pressure by almost 30 to 142.8 mm Hg (Figure 2A).

### Metabolic Cage Analyses

On 0.3% Na diet, Hsd2KO animals were clearly polydipsic and polyuric (from 7 weeks of age; data not shown). Both urinary albumin and calcium excretion levels were significantly higher in Hsd2KO samples than controls (Figure S11). This probably reflected the exposure to high blood pressure because both albumin and calcium levels were decreased on the 0.03% diet.

On 0.3% Na diet, urinary Na/K ratio was slightly higher in Hsd2KO animals and a significantly higher percentage of sodium intake was secreted by the kidney (Table), the balance being excreted through the gut.^19,20^ On transfer to 3% Na^+^ diet, increases in water intake and urine production were greater in Hsd2KO than in WT rats (Table). Hsd2KO rats ate less of the 3% Na diet and achieved a lower Na/K ratio. WT (and Hsd2+/−) animals maintained a normal sodium balance by increased urinary sodium excretion. Hsd2KO animals were not able to maintain an appropriate natriuretic response on a 3% Na diet. After 3 days on this diet, they developed diarrhea, urinary Na/K ratios fell, and the study was terminated.

**Table.**
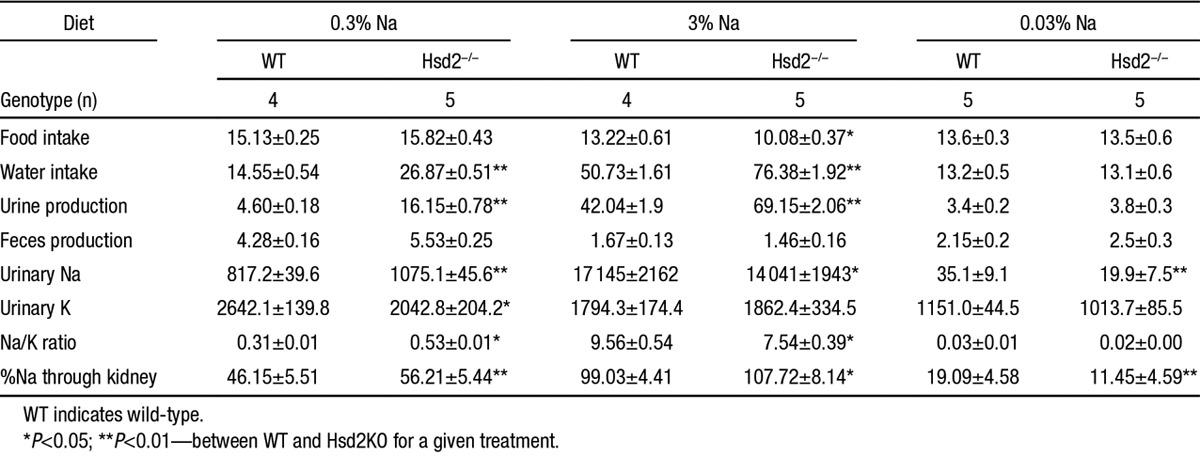
Metabolic Analysis of WT and Hsd2KO Animals on 0.3%, 3%, and 0.03% Na Diets

On transfer from a 0.3% Na to a 0.03% Na diet, water consumption, urine production and sodium excretion dropped to broadly WT levels (Table). The drop in urinary Na^+^ and Na/K ratio was more acute in Hsd2KO rats than in controls (Figure 2B and 2C), and cumulative sodium balance in WT but not Hsd2KO rats was negative across the first few days (Figure 2D). On the 0.03% Na diet, homozygotes excreted a significantly lower percentage of sodium via the kidney (Table).

### Hormone Measurements

**Figure 3.**
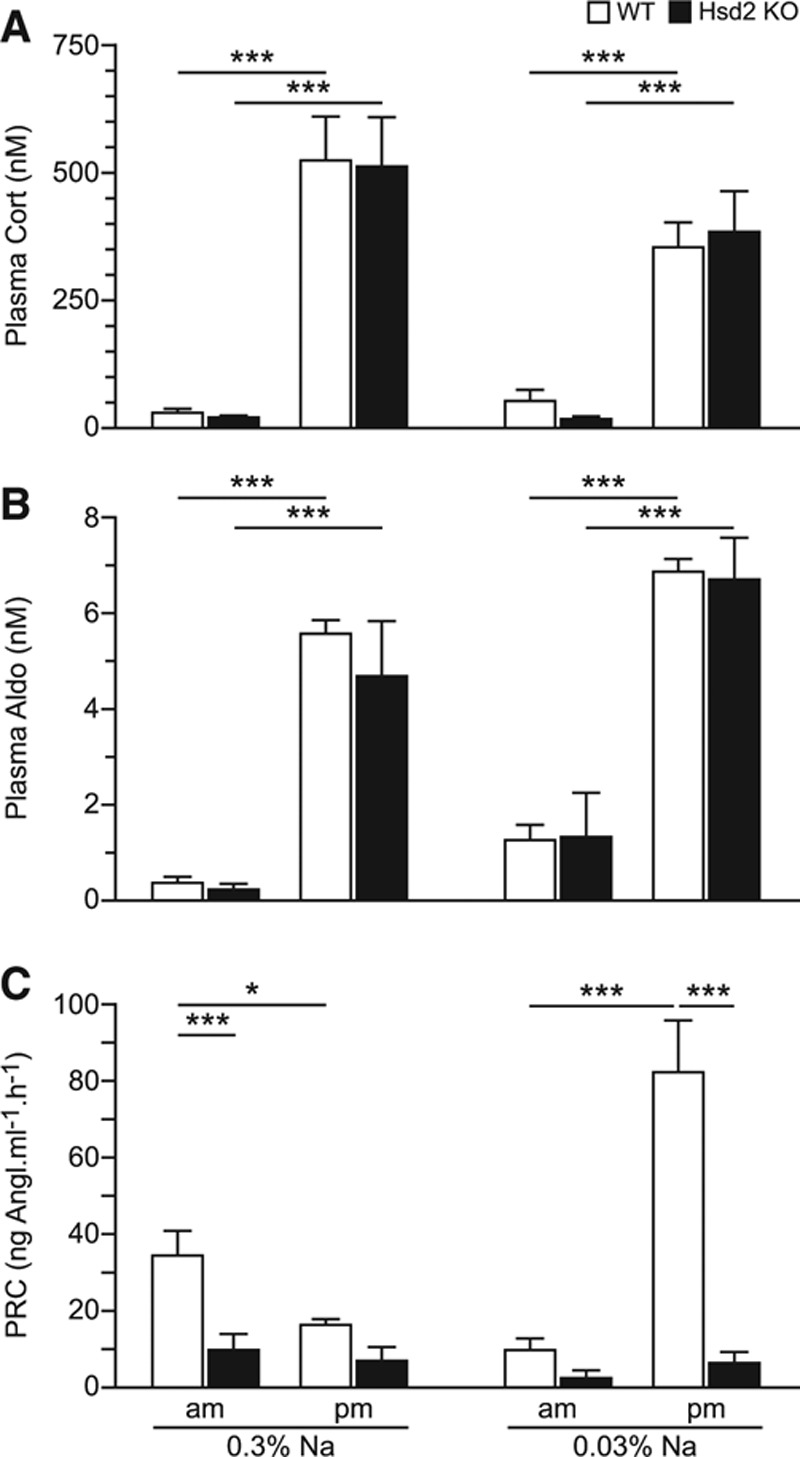
Graphs showing morning (7:00 am) and evening (7:00 pm) plasma concentrations of (**A**) corticosterone, (**B**) aldosterone, and (**C**) renin on 0.3% and 0.03% Na diets. PRC indicates plasma renin concentration; and WT, wild-type. *P<0.05; ***P<0.001.

Plasma corticosterone and aldosterone levels, taken at 7:00 am and 7:00 pm, showed expected diurnal variation^21,22^ on both the 0.3% and 0.03% Na diets, and did not vary significantly from control levels (Figure 3A and 3B). Renin levels, however, were lower than WT levels at 7:00 am and 7:00 pm on 0.3% Na diet and were significantly suppressed in the evening on the 0.03% Na diet (Figure 3C). Two-way ANOVA of aldosterone/plasma renin concentration shows that Hsd2KO values are ≈8-fold higher than WT (178±68.5 versus 20.3±5.1) due entirely to the suppression of plasma renin concentration. Diet and time did not contribute significantly to variation and showed no significant interactions with genotype.

### Glucose Tolerance Test

**Figure 4.**
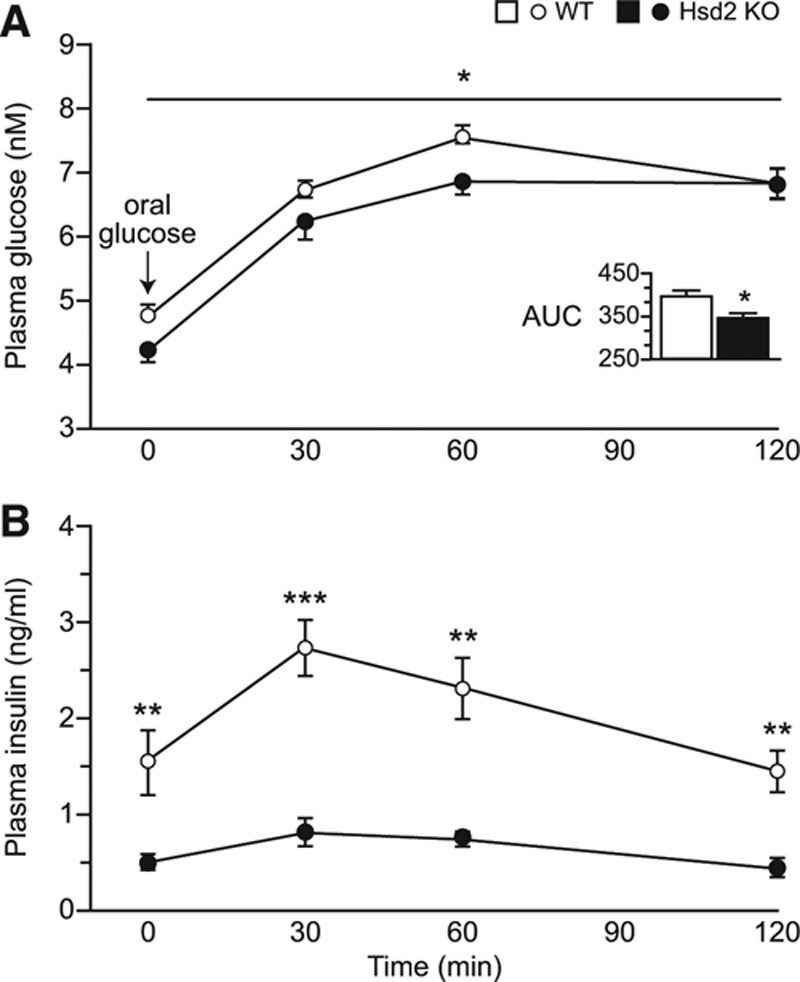
Graphs showing plasma levels of: (**A**) glucose and (**B**) insulin, during glucose tolerance test (*P<0.05; **P<0.01; ***P<0.001). Inset shows respective areas under the curves (AUC). WT indicates wild-type.

Increases in plasma glucose in response to an oral challenge were similar in Hsd2KO and WT animals, but levels overall were lower in Hsd2KO rats, indicating that homozygotes were more sensitive to insulin (Figure 4A). Indeed, plasma insulin levels during the test were significantly lower in the Hsd2KO animals (Figure 4B) at all time points. Homeostasis model assessment measurements for fasted rats are WT: 11.75±1.98; KO: 4.12±0.63, confirming greater insulin sensitivity (*P*<0.005).

## Discussion

We made a rat Hsd2 null model to capitalize on the phenotyping possibilities of this species. ZFN targeting proved to be an effective method for producing an allelic series—6 deletions and 1 insertion event—in the *Hsd11b2* gene. The optimum mRNA concentration for founder production was 3.125 ng/µL. The majority of targeting events (4 bp or 16 bp deletions and 1 bp insertion) brought a TGA stop codon into frame in exon 2. The 2 larger deletions (123 bp and 332 bp) removed part or all of exon 2, respectively. Although aberrantly spliced transcripts were observed in the Hsd2KO animals carrying the 123 bp deletion, no enzyme activity, and no full-length protein on Western analysis were observed. This suggests that the weak immunohistochemical signal seen in kidney sections with anti-Hsd11b2 antibody represents nonfunctional protein.

Hsd2KO rats were born in normal Mendelian ratios and were not disadvantaged neonatally. Reduction in adult body weight was due, in part, to decreased adiposity. Nevertheless, kidney and heart weights were higher, possibly resulting from chronic exposure to high blood pressure. It is not known whether the fibrotic damage and mild inflammation seen in the hearts of Hsd2 knockouts at 6 months of age is caused before or because of hypertensive damage. Homozygous rats exhibited several signs of mineralocorticoid excess. Blood pressure was already high on a 0.3% Na diet and was reduced significantly by 0.03% Na diet. There was evidence of hypokalemia (data not shown) and plasma renin was suppressed even despite a 0.03% Na diet.

Increased kidney size reflected hyperplasia (but not hypertrophy) of the DCT in young adult Hsd2KO rats. Subtle signs of kidney damage included Kim-1 expression and macrophage infiltration. Increases in interstitial cells and glomerulosclerotic and fibrinoid necrotic changes were evident in older animals, but unlike older Hsd2KO mice, the renal medulla did not show overt signs of atrophy. As in human patients and the mouse SAME model, Hsd2KO rats exhibit overt polydipsia and polyuria. In mice, failure to concentrate urine has been attributed to reduced expression of aquaporins progressing in older animals to a form of nephrogenic diabetes mellitus insipidus^23^ linked to the degeneration of the renal medulla.^14^ In Hsd2KO rats polyuria does not worsen with age consistent with a lack of effect on renal medulla. However, although the polyuria is significantly worse than WT rats on a 3% Na diet, there is no genotypic difference in water intake or urine volume on 0.03% Na diet. Given that blood pressure and urinary excretion patterns of Hsd2KO and WT rats follow similar trends in relation to dietary sodium intake, it is possible that pressure natriuresis differences^24^ explain urine volume variation both between genotypes and in response to dietary manipulation.

Unlike the mouse SAME model,^12^ the antinatriuretic/kaliuretic activity of apparent mineralocorticoid excess was not reflected in urinary Na/K ratios or increased plasma volume. Indeed Na/K was higher, not lower, in Hsd2KO rats and haematocrit values suggested volume contraction rather than expansion. Volume contraction has been noted in other models of steroid hormone excess and mineralocorticoid-dependent changes in urinary electrolyte excretion are only transient during adaptation from one steady state to another. Evidence suggests that the null rat is primed to excrete a greater proportion of ingested salt through the kidney on 0.3% diet, is unable to adequately cope with salt load on the 3% diet, and is capable of rapidly shutting down kidney Na excretion on the 0.03% diet as blood pressure drops. These kidney adaptive responses may be linked to fecal sodium and potassium excretion because in normal rats, colonic and renal 11βHSD2 activity are differentially expressed in response to dietary sodium manipulation.^19,20^ In addition, there could be an autonomic nervous system component because small changes in plasma Na (2 or 3 mmol/L) can exert profound effects on the sympathetic nervous system.

Predictably, dietary sodium restriction in WT rats was associated with increased plasma aldosterone and a marked increase in renin at night time.^22^ However, unlike SAME in humans and mice, plasma aldosterone was not suppressed in Hsd2KO rats. Moreover, zona glomerulosa cells of Hsd2KO rats were hypertrophied, albeit slightly, compared with WT cells. This was unexpected because plasma renin and potassium concentrations, the main trophic factors for aldosterone synthesis, were both markedly reduced. The third trophic factor, adrenocorticotropic hormone, is an unlikely candidate to explain the lack of aldosterone suppression because diurnal changes in corticosterone and zona fasciculata cell size were unaffected by genotype and zona reticularis cells were smaller in Hsd2KO rats. There are, however, many other known factors (eg, dopamine, serotonin, local adrenal renin angiotensin system, renal clearance of aldosterone, or atrial natriuretic hormone) that might be involved. Given evidence of vascular volume contraction, it would seem likely that atrial natriuretic hormone is suppressed in Hsd2KO rats, thereby removing a tonic inhibitory effect on aldosterone synthesis^25^ and contributing to the aldosterone escape.

The metabolic phenotype of reduced adiposity and increased insulin sensitivity suggest glucocorticoid activity is reduced in Hsd2KO rats. This argues against the idea that Hsd11b2 activity limits access to GR in the same way that mineralocorticoid receptor is protected and highlights the importance of the isozyme, Hsd11b1, in glucocorticoid-regulated tissues, such as the liver, pancreas, and adipose tissues. In the absence of Hsd11b2, there is no substrate for Hsd11b1 and, therefore, no local regeneration of active hormone^26^ in tissues where glucocorticoids influence glucose and lipid metabolism. The lack of macrophage infiltrate in mesenteric fat pads reflected the favorable metabolic phenotype. Both improved glucose tolerance and insulin sensitivity in adipocytes and liver have been demonstrated in the mouse Hsd11b1^−/−^ model,^27,28^ whereas Hsd11b1 inhibition has previously been reported^29^ to specifically reduce fat accretion in the mesenteric fat pad.

Interestingly, the adrenal does not show the hypertrophied phenotype of Hsd11b1 deficiency,^30^ presumably because reduced clearance of glucocorticoids by Hsd11b2 is counterbalanced by reduced regeneration via Hsd11b1. This idea of opposing effects of Hsd11b isozymes could be extended to other tissues. For example, even in the kidney, where the SAME phenotype is paramount, it should be noted that Hsd11b1 is expressed in proximal tubules where GR-mediated effects on fluid and electrolyte balance have been described.

In summary, we have developed a rat SAME model exhibiting many classic characteristics of the Human SAME patient. In addition, the effective chronic loss of key Hsd11b1 and Hsd11b2 activities, aldosterone escape and a protective metabolic profile, superimposed on salt-sensitive hypertension, provides scope for extended mechanistic characterizations afforded by this species.

### Perspectives

With the advent of efficient, relatively cost-effective techniques for targeting genome modifications in the rat, including ZFNs, Talens, and the Crispr-Cas9 system, we can now begin to fully capitalize on the wider range of experimental possibilities available for this species. The rat SAME model presents with an intriguing and complex mix of protective metabolic effects superimposed on a background of chronic salt-sensitive hypertension, which further define the subtleties of Hsd11b2 loss of function in SAME.

## Acknowledgments

We wish to acknowledge the technical assistance of G. Brooker, J. Noble, and A. Peter; bioinformatics assistance from Dr Manning; the Genetic Intervention and Screening Technologies unit, University of Edinburgh for microinjections; Wellcome Trust Mass Spectrometry Core for steroid analysis.

## Sources of Funding

We are grateful for funding from the British Heart Foundation Centre of Research Excellence Award (RE/08/001/23904) and from the European Union consortium EURATRANS (HEALTH-F4-2010–241504).

## Disclosures

None.

## Supplementary Material

## Novelty and Significance

### What Is New?

- The rat syndrome of apparent mineralocorticoid excess model reported here is extraordinary because of the normal aldosterone levels seen in the context of apparent mineralocorticoid excess, suggesting aldosterone escape. The metabolic sequelae of Hsd11b2 knockout reflect the concomitant effective knockout of Hsd11b1 activity.

### What Is Relevant?

- The interplay between reduced function of Hsd11b2, Hsd11b1, and aldosterone could inform certain cases of essential hypertension.

### Summary

The rat syndrome of apparent mineralocorticoid excess model presents with an intriguing and complex mix of protective metabolic effects superimposed on a background of chronic, salt-sensitive hypertension.
