# Supplementary material for: Mineralocorticoid Excess or Glucocorticoid Insufficiency: Renal and Metabolic Phenotypes in a Rat Hsd11b2 Knockout Model
Source: Hypertension. 2015 Aug 12;66(3):667–73. doi: 10.1161/HYPERTENSIONAHA.115.05262 (PMC4847935; doi:10.1161/HYPERTENSIONAHA.115.05262)
Supplement: Supplementary file 1 [file hyp-66-667-s001.pdf]

MINERALOCORTICOID EXCESS OR GLUCOCORTICOID INSUFFICIENCY: RENAL AND METABOLIC PHENOTYPES IN A RAT HSD11B2 KNOCKOUT MODEL

Dr. Linda J Mullins, Dr. Christopher J Kenyon, Dr. Matthew A Bailey, Dr. Bryan R. Conway, Dr. Mary Diaz and Prof. John J Mullins

Molecular Physiology Laboratory,

University of Edinburgh/ BHF Centre for Cardiovascular Science,

Queen's Medical Research Institute, University of Edinburgh, Edinburgh, UK

Short title: SAME rat model

Corresponding Author's address,

Dr Linda J Mullins, University of Edinburgh/BHF Centre for Cardiovascular Science, Queen's Medical Research Institute, 47, Little France Crescent, Edinburgh EH16 4TJ

Tel: +44(0) 131 242 6720; Fax: +44 (0) 131 242 6782

e-mail: Linda.mullins@ed.ac.uk

Supplementary Materials and methods

Animal experiments

All experiments were approved by the local ethics committee and conducted in accordance with UK Home Office regulations and the Animals (Scientific Procedures) Act 1986. Animals were housed in standard cages, maintained on a 12 h light/dark cycle (7.00am to 7.00pm), and given free access to water and standard rat chow. Fischer (F344IcoCrl) rats were supplied by Charles River Laboratories. Groups of 4 to 8 adult male rats were used in metabolic cage studies, for telemetric analysis and for glucose tolerance test. For single cell embryo harvesting, females were superovulated by injection of pregnant mare's serum gonadotrophin (Intervet; 20iu) at 9a.m. and then Human chorionic gonadotrophin ('Chorulon' Intervet; 30iu) 48 h later. They were then mated that evening. Females were plug-checked the following morning and embryos were flushed.

ZFN design strategy and Generation of Targeted knockout animals

Targeting zinc-finger nuclease mRNAs, directed to exon 2 of the *Hsd11b2* gene and incorporating obligate heterodimer FokI domains, were designed with the help of CompoZr Custom ZFN Service, (Sigma Life Science). The ZFN binding sites and spacer sequence are given in **Table S2**. The ZFN mRNAs (diluted from 1 to 10ng/μl in microinjection buffer; 1mM Tris HCl, 0.25mM EDTA) were microinjected into the pronucleus of 1-cell embryos, which were returned to pseudo-pregnant females at the two-cell stage. Three pairs of PCR primers (**Table S2**) were designed for screening the progeny. Using a crude DNA preparation from tissue biopsy (as described by Geurtz et al.,[1]), the first primer pair (F1-R1) generated a 332bp fragment spanning the target area, which was denatured, re-annealed, cut with Surveyor nuclease (Transgenomic, Omaha, NE), and analyzed by gel electrophoresis. This identified progeny carrying small insertions or deletions (indels). PCR products generated using the other primer pairs were compared with parental products (630bp and 1.68kb respectively) to screen for larger deletions.

To determine the accurate size of ZFN indel modifications, PCR products were generated using Accuprime HiFi polymerase (Invitrogen), which adds adenosine to the 3' end. PCR products were then cloned, using the Topo-TA cloning kit (Invitrogen) and their sequence compared with parental sequence, using the respective PCR primers as sequencing primers. The Δ123 strain (see Fig S1B) was genotyped using primer pair 2, which gave ~410bp product for Hsd2-/- animals.

Tissue processing & Immunohistochemistry

Tissues were harvested into RNA later, frozen on dry ice, fixed in 4% PFA ON at 4°C, or fixed in formalin overnight, as necessary. For perfusion fixation, animals were anaesthetized under isoflurane, and the infra-renal aorta was cannulated to allow retrograde perfusion of 150 ml fresh 4% PFA in PBS, pH 7.4. Tissues were then removed, trimmed and placed in 4% PFA overnight at 4°C, followed by 70% ethanol. All fixed tissues were embedded in paraffin wax and 5μ sections cut.

Sections were de-waxed, hydrated and heated in Novocastra Bond Epitope Retrieval Solution 1 pH 6.0 (ERI, Leica), and target antigens were detected using a Leica BOND-MAX robot. Primary antibodies were: sheep anti-Hsd11b2 (Millipore,

AB1296; 1:500); rabbit anti-NCC (Millipore, AB3553; 1:500); rabbit anti-Hsd11b1 (Abcam AB39364; 1:250); mouse anti-ED-1 (AbD Serotec; 1:100); goat anti-Kim1 (R&D sytems; 1:100). Immuno-reaction was visualized using the Bond polymer refine detection kit (3S9800; Leica). Some sections were stained with H & E stain or Masson's Trichrome. Images were obtained using an Olympus BX51 and Q-capture Pro or an Olympus AX70 and Axiovision REL4.8. For ED1 analysis, antigen-expressing cells were counted per field (five random fields (x20 magnification) per animal).

Quantitation of distal tubular structures in Hsd2KO rats

Sections were taken from adult WT and Hsd2KO 4% paraformaldehyde-fixed kidneys and were stained for NCC, Hsd11b2 or Hsd11b1. All glomerular cross sections, and all tubular cross sections (all cortical nephron segments) were counted in a given field. Respective antigen-expressing tubules were then counted as a percentage of the total, and the number of antigen-expressing tubular cell nuclei per field were counted in order to ascertain the average nuclei count per tubule cross-section.

For Kim1 analysis the percentage of antigen-expressing cortical nephron segments were expressed as a percentage of all tubular cross sections (five random fields (x10 magnification) per animal were counted).

Glomerular number was ascertained by a modification of the method of Habib et al. [2]. Under anaesthesia, (isoflurane) Alcian Blue AX (5% in isotonic saline; Sigma Chemical Company) was infused over 30s (0.25ml/100g body weight), and after 5 min, a second dose was administered. After a further 5 min, kidneys were harvested, de-capsulated, minced in a small volume of 1% ammonium chloride, incubated (5 min, RT), then sieved through a 40um strainer and incubated in 50% HCl (6N) in a shaking water bath at 37°C. After 90 min the sample was vortexed vigorously until tissue pieces were broken down. Following centrifugation (3000rpm for 10 min), the supernatant was discarded and the pellet resuspended in 50 ml distilled water. Finally, 30 replicates (10µl each; removed after gentle resuspension) were counted, and the total number of glomeruli was extrapolated per kidney.

Corticosterone and 11-dehydrocorticosterone analyses by Mass spectrometry

Blood was collected into EDTA, mixed thoroughly and following centrifugation; plasma aliquots were immediately frozen at -80°C. On thawing, steroids were extracted from plasma (1mL) enriched with internal standard (epi-corticosterone (500ng; Steraloids, US) using chloroform (1:10). The organic phase was reduced the dryness and reconstituted in mobile phase (water: acetonitrile, 70:30, with 0.1% formic acid). Steroids were eluted (0.5mL/min) isocratically from a Sunfire column (C18 3.5um, 2.1 x 100mm) at 10C and the following transitions (Declustering potential, Collision Energy, Cell Exit Potential) monitored; A 345- 121.1 (51, 33, 8V), B 347-91.1 (66, 69, 8V), with collision gas Medium, Ion source 5500V, source temperature 450C.

Metabolic studies

Male rats (groups of 4 to 5; 22 weeks old) were housed individually in metabolic cages and after an acclimatization period (four days), with standard rat chow and water *ad libidum,* urine samples were collected for 5- 7 days to establish a baseline.

Diet was then changed to either high salt (3% Na) diet or a low salt diet (0.03% Na) for an additional 7 to 10 days. Food and water consumption were measured throughout.

Urine analyses

Urinary sodium and potassium ion levels were measured using a BWB-1 Flame Photometer (BWB Technologies UK Ltd) according to the manufacturers instructions. Urine samples were diluted 100-fold and read against standard curves prepared for Na^+ and K^+ respectively. Urine samples were also analyzed for albumin, calcium and creatinine. Albumin: creatinine and calcium: creatinine ratios were calculated in order to correct for urine volume.

Glucose tolerance test and insulin determination

Adult rats (groups of 8; 23 to 25 weeks old) were fasted overnight and then injected intraperitoneally with 2g/kg D-glucose (50% stock solution in saline). Blood samples were taken by tail venesection into EDTA-micro-tubes (Sarstedt, Leicester, UK) at 0 min (before injection and within 1 min of disturbing the cage) and at 30, 60 and 120-min intervals after glucose administration. Blood glucose was measured using a One-Touch Ultrasmart blood glucose meter (Lifespan, Scotland). Plasma was stored at -20°C. Insulin concentration was measured by ELISA (Crystal Chem Inc, Chicago, IL).

Glucocorticoid, aldosterone and renin measurements

Plasma corticosterone, aldosterone and renin concentrations were measured by radioimmunoassay as previously described[3-5]

Telemetric analyses

Adult rats (4 to 6 per group; 26 to 30 weeks old) were monitored by telemetry. Systolic and diastolic blood pressures, together with mean arterial blood pressure (MABP), heart rate, pulse pressure and activity were monitored using rat telemetry devices (TA11PA-C40; Data Sciences International, St. Paul, Minnesota, USA), with the catheter placed below the bifurcation of the renal arteries, in the abdominal aorta, under anaesthesia (isoflurane). Vetegesic was administered post surgery. Data was collected at 1000 Hz for 5 min, every hour. Animals were allowed to recover from surgery for 7 days before the collection of baseline data and subsequent dietary changes.

Statistical analyses

Data are presented as the mean +/- SEM. Variables (genotype and diet) were compared by performing two-way ANOVA using Prism6. Mean values were compared using the Student's t-test. A $P<0.05$ was considered to be statistically significant.

References

1. Geurts AM, Cost GJ, Remy S, Cui X, Tesson L, Usal C, Menoret S, Jacob HJ, Anegon I and Buelow R. Generation of gene-specific mutated rats using zinc-finger nucleases. *Methods Mol Biol*. 2010;597:211-225.
2. Habib S, Zhang Q and Baum M. Prenatal programming of hypertension in the rat: effect of postnatal rearing. *Nephron Extra*. 1:157-165.
3. Kenyon CJ, Panarelli M, Holloway CD, Dunlop D, Morton JJ, Connell JM and Fraser R. The role of glucocorticoid activity in the inheritance of hypertension: studies in the rat. *J Steroid Biochem Mol Biol*. 1993;45:7-11.
4. Al-Dujaili EA and Edwards CR. The development and application of a direct radioimmunoassay for plasma aldosterone using 125I-labeled ligand--comparison of three methods. *The Journal of clinical endocrinology and metabolism*. 1978;46:105-113.
5. Millar JA, Leckie BJ, Morton JJ, Jordan J and Tree M. A microassay for active and total renin concentration in human plasma based on antibody trapping. *Clinica chimica acta; international journal of clinical chemistry*. 1980;101:5-15.

Table S1 - ZFN-mediated gene disruption after pronuclear microinjection

ZFN mRNA dose (ng/µl)	#surviving embryos(%)	# born (%)	mutants born
10	90 (60)	4 (4)	0
3.125	92 (70)	24 (26)	4
2.5	68 (77)	15 (22)	0
1	25 (83)	6 (24)	0
	TOTAL	48	4

Table S2– ZFN target sequence and PCR primers used to screen indels

target/primer	sequence	comments
ZFN Target sequence	ACTGCGTGCCCGATGttccccTCGCCTGAAGCTGC	double strand cut (lowercase)
ZFN Primer F1	GAAGGGAGGGCTGAGTATCC	deletions up to 332 bp
ZFN Primer R1	CTGCCTCCTTCACACTCTCC	
ZFN Primer F2	CCTCTGACTTAGGCTGAGGACAAGTTGG	deletions up to 630 bp
ZFN Primer R2	GCCACTACCATGTTGAGGCCAGC	
ZFN Primer F3	GAGTGAGGAAGCTGGGCTG	deletions up to 1.68 kb
ZFN Primer R3	CCACACTACATGCTCAGACCAC	

Table S3: RT-PCR primers spanning exon junctions

for	rev	exon span	WT amplicon	Hsd2-/- amplicons
gccctggtgctctagaactg	tgttaaccagaccccacagg	2 to 3	147 nt	40 nt
caaggggacgtattgtgacc	ctgccaagcaggggtatg	3 to 4	65 nt	65 nt<br>172 nt
ggggtatcaaggtcagcatc	tcccagaggttcacattagtca	4 to 5	72nt	72 nt<br>229 nt
gccctggtgctctagaactg	ctgccaagcaggggtatg	2 to 4	342 nt	235 nt<br>340 nt
gccctggtgctctagaactg	tcccagaggttcacattagtca	2 to 5	477 nt	370, 477,<br>527, 634 nt
caaggggacgtattgtgacc	tcccagaggttcacattagtca	3 to 5	200 nt	200nt<br>307, 465 nt

(A)

```
                                        Z F N R
g A A C T G C G T G C C C G A T G t t c c c c T C G C C T G A A G C T G C T g
c T T G A C G C A C G G G C T A G a a c c c c A G C G G A C T T C G A C G A c
        Z F N L
```

(B)

```
allele
WT    g A A C T G C G T G C C C G A T G t t c c c c T C G C C T G A A G C T G C T g
D4    g A A C T G C G T G C C C G A T - - - - c c c T C G C C T G A A G C T G C T g
D16   g A A C T G C G T G C C - - - - - - - - - - - - - - - - T G A A G C T G C T g
D123  g A A C T - - - - - - - - - - - - - - - - - - - - - - - - - - - - - - - - - -
D332  - - - - - - - - - - - - - - - - - - - - - - - - - - - - - - - - - - - - - -
```

Figure S1: (A) ZFN target site (lower case) showing sequences of ZFNL and ZFNR (highlighted in yellow. (B) Insertions and deletions identified in ZFN-targeted founder #6. (Note TGA (red) brought into frame with 4bp and 16bp deletions)

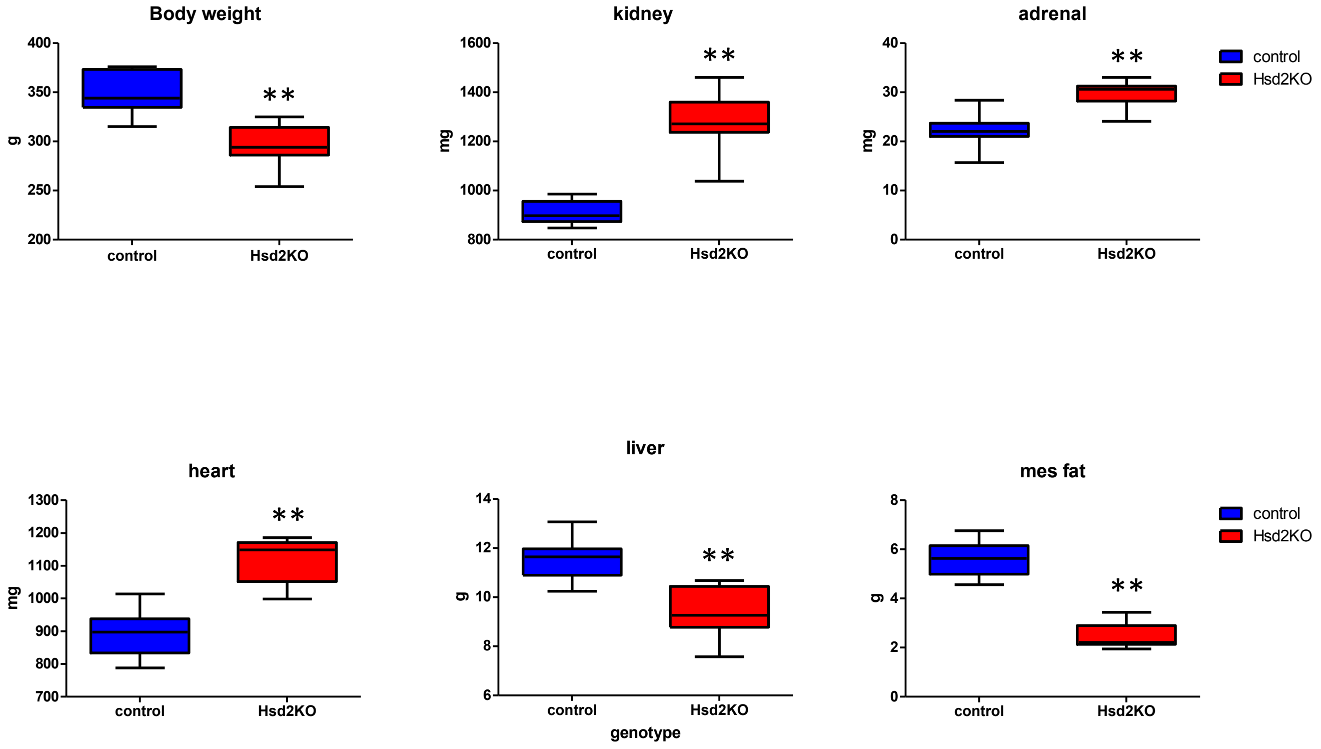

Figure S2: Relative body weight, kidney, heart, adrenal, liver and mesenteric fat pad weights of WT (blue) and Hsd2KO (red) animals. (**P<0.01)

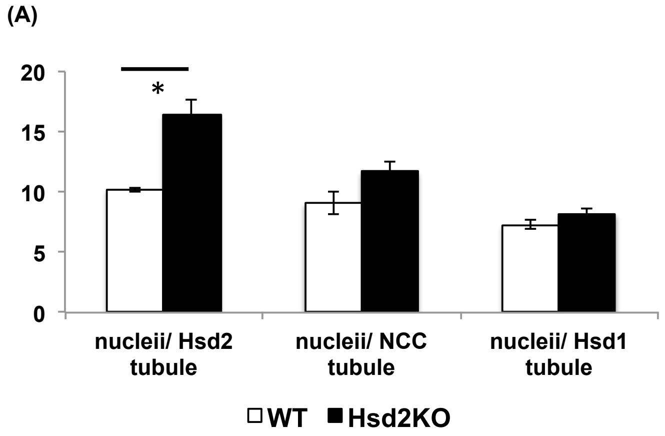

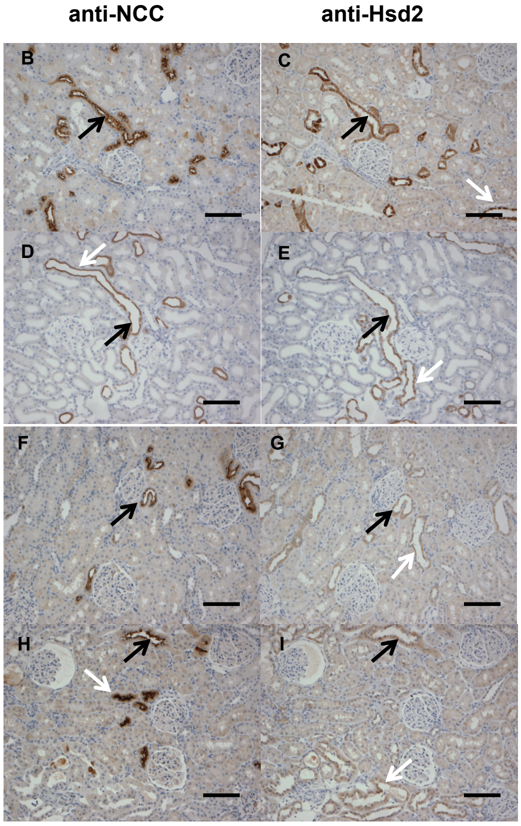

Figure S3: (A) Relative hyperplasia of the distal nephron, as measured by number of nuclei in Hsd2- (and NCC-) expressing tubules but not Hsd1-expressing (proximal) tubules. Pairs of serial sections from (B-E) WT and (F-I) Hsd2KO kidneys stained with anti-NCC or anti-Hsd2, showing tubules with co-localization of antigens (black arrows) and tubules with unique antigen expression (white arrows); (all 10x magnification; bar – 100µm)(*P<0.05)

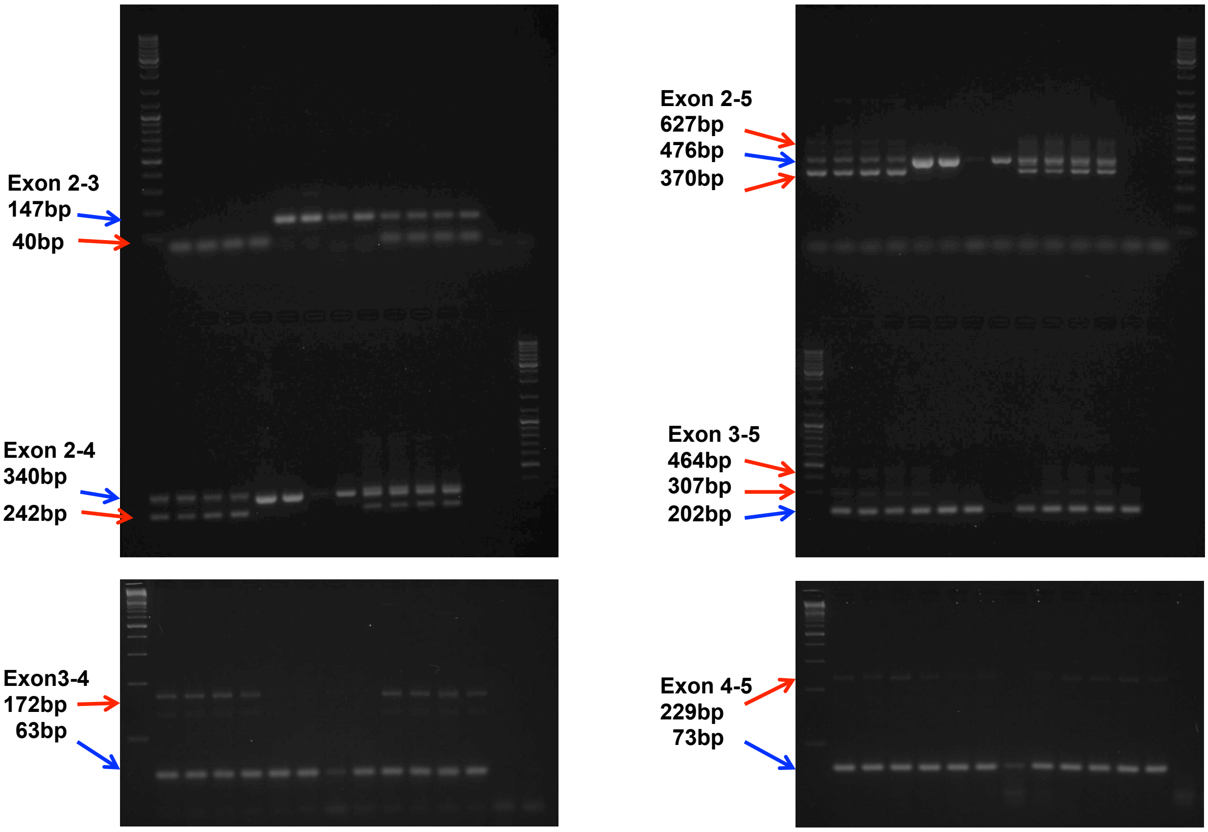

Figure S4: RT-PCR analysis across exon borders. In all gels: samples 1-4: Hsd2KO; 5-8: WT; 9-12: Hsd2KO spiked with WT; marker tracks use GeneRuler. A) upper loading – RT-PCR products from exon 2 to exon 3 screen; lower loading – exon 2 to exon 4 screen; B) exon 3 to exon 4 screen; C) upper loading – exon 2 to exon 5 screen; lower loading - exon 3 to exon 5; D) exon 4 to exon 5 screen. Blue arrows indicate WT RT-PCR products. Red arrows indicate RT-PCR products from alternative splice variants

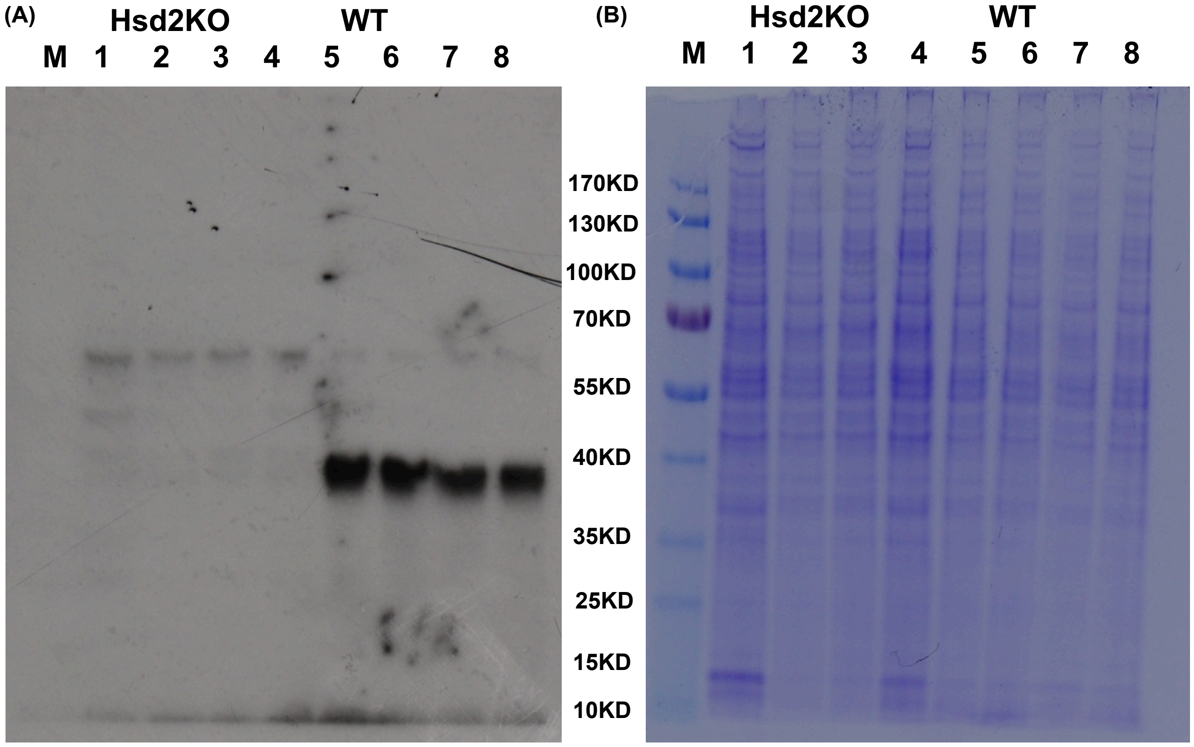

Figure S5: Western blot of proteins extracted from kidney and stained with anti-Hsd11b2 antibody. A) tracks 1 to 4 Hsd2KO; tracks 5 to 8 WT; M – protein ladder. B) As per A) but stained with Coomassie Blue

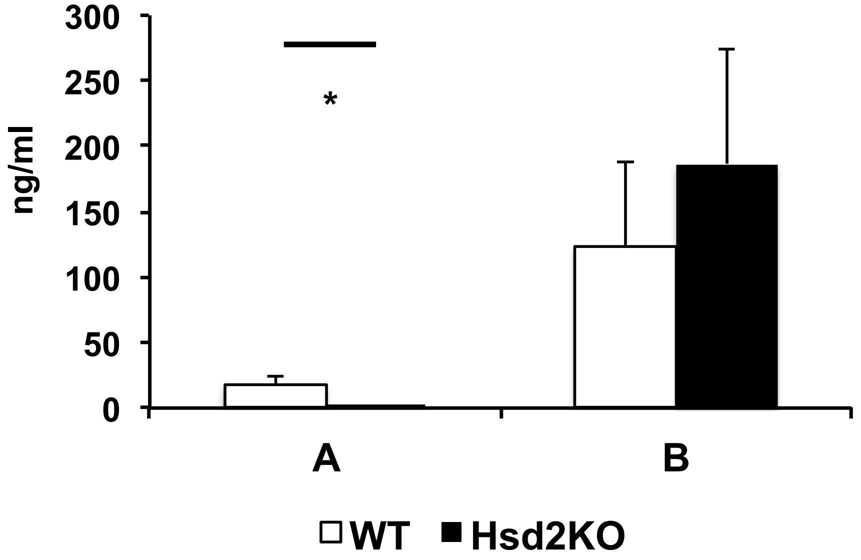

Figure S6: Plasma glucocorticoids (product A: 11-dehydrocorticosterone; product B: corticosterone) measured by Mass Spectrometry (*P<0.05)

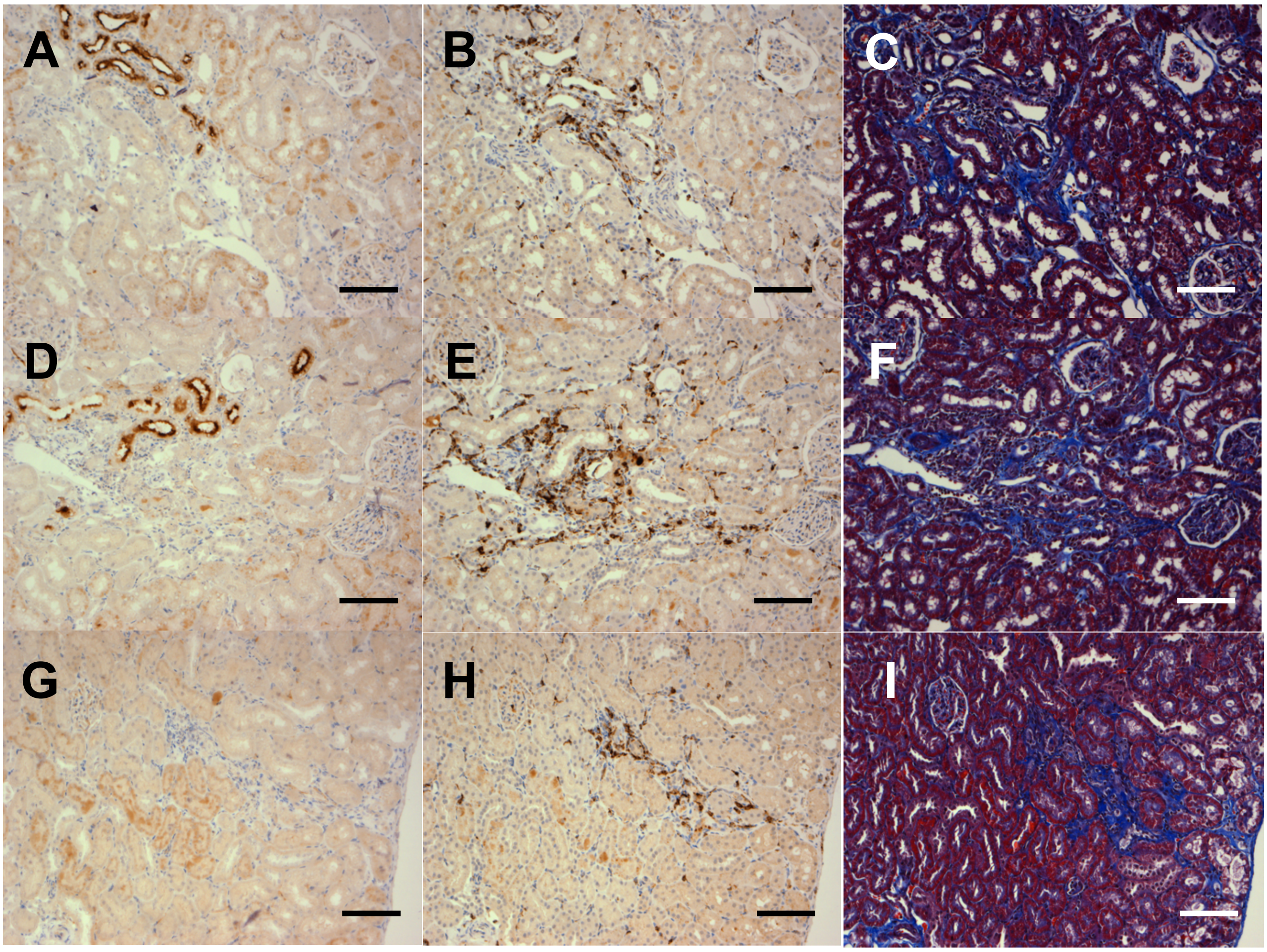

Figure S7 – serial kidney sections from Hsd2 null (A-F) and wild type (G-I), 26 week-old animals, stained with Kim-1 (A,D,G), ED1 (B,E,H) and Masson's Trichrome (C,F,I). (All 10x magnification; bar = 100µm)

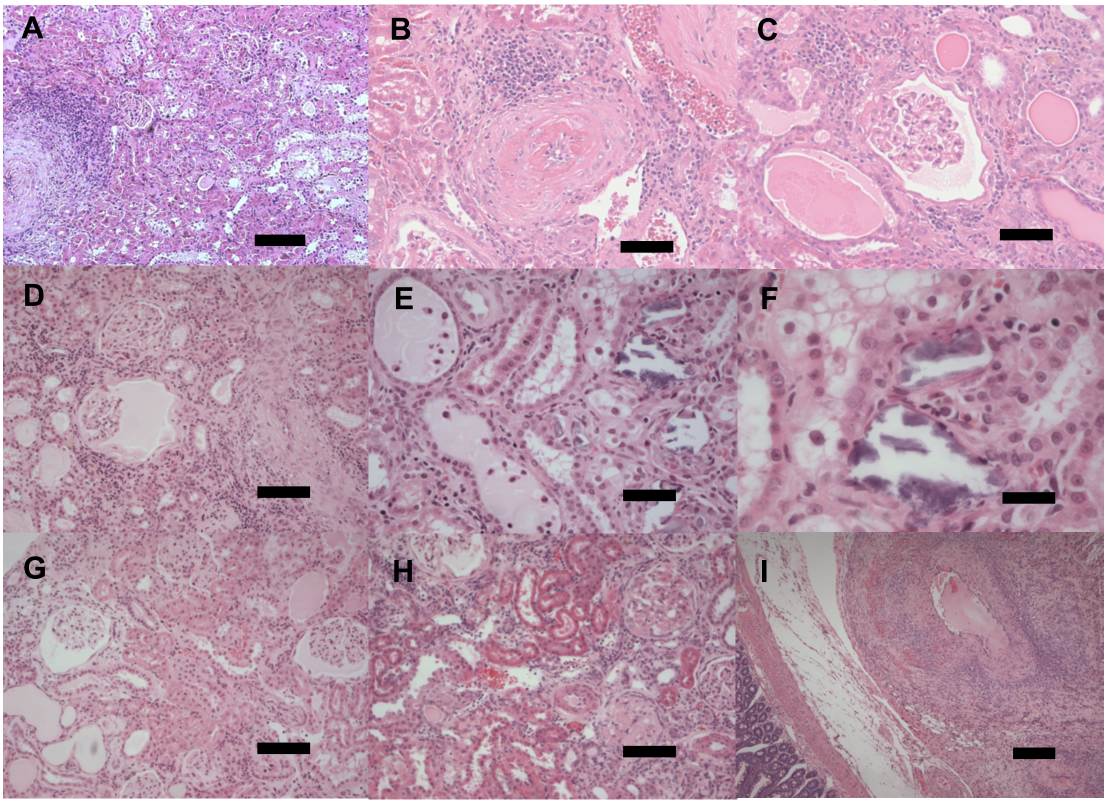

Figure S8: Aged Hsd2KO animals (9-12 months old). Panels A to F – male kidney sections showing fibrinoid necrosis (A, B), amorphous protein in Bowman's capsule (C, D), sloughing of cells in tubules (E) and calcium crystals (F); Panels G to H (kidney) and panel I (mesenteric artery) from pregnant female displaying pan arteritis. H&E staining. (A-C, E 20x magnification, bar=50µm; D, G-H – 10x, bar=100µm; F – 40x, bar=25µm; I – 4x, bar=100µm)

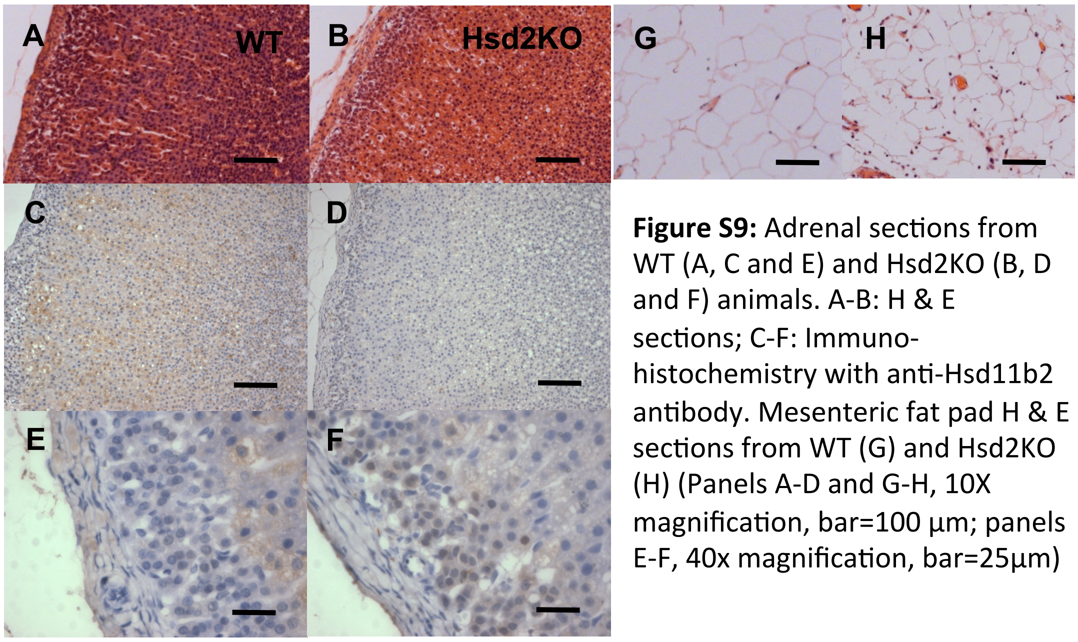

Figure S9: Adrenal sections from WT (A, C and E) and Hsd2KO (B, D and F) animals. A-B: H & E sections; C-F: Immuno-histochemistry with anti-Hsd11b2 antibody. Mesenteric fat pad H & E sections from WT (G) and Hsd2KO (H) (Panels A-D and G-H, 10X magnification, bar=100 µm; panels E-F, 40x magnification, bar=25µm)

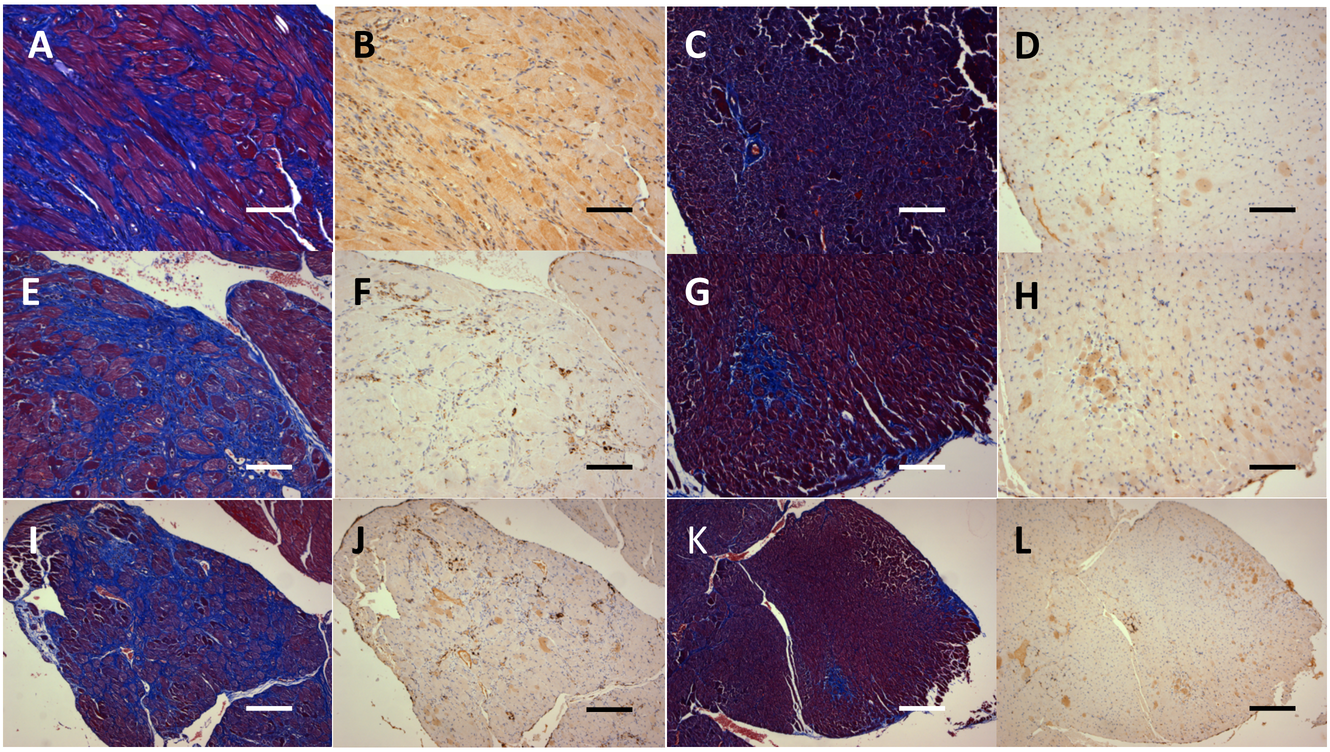

Figure S10: serial heart sections from Hsd2 null animals (columns 1 and 2) and controls (columns 3 and 4) stained with Masson's Trichrome (A, C, E, G, I, K) or anti-ED1 antibody (B, D, F, H, J, L). (All 20x magnification; Bar = 100μm)

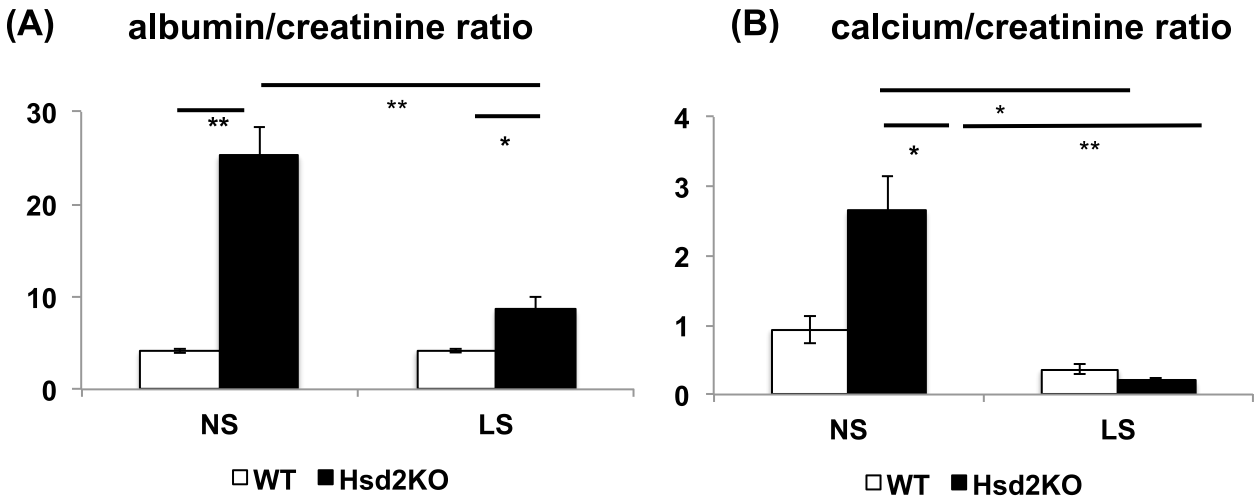

Figure S11: Urine samples, collected on 0.3% Na (NS) and 0.03% Na (LS) diets, were analyzed for albumin and calcium, which were expressed as a ratio to creatinine (a.u.) (*P<0.05;**P<0.01)
